# From Water Source to Tap of Ceramic Filters—Factors That Influence Water Quality Between Collection and Consumption in Rural Households in Nepal

**DOI:** 10.3390/ijerph15112439

**Published:** 2018-11-01

**Authors:** Regula Meierhofer, Carola Bänziger, Sandro Deppeler, Bal Mukund Kunwar, Madan Bhatta

**Affiliations:** 1 Department of Sanitation, Water and Solid Waste for Development (Sandec), Swiss Federal Institute of Aquatic Science and Technology (Eawag), Ueberlandstrasse 133, 8600 Dübendorf, Switzerland; carolaba@student.ethz.ch (C.B.); deppeler.sandro@gmail.com (S.D.); 2 Helvetas Swiss Intercooperation Nepal, Dhobighat, Lalitpur G.P.O. Box 688, Nepal; Bal.Kunwar@helvetas.org (B.M.K.); Madan.Bhatta@helvetas.org (M.B.)

**Keywords:** drinking water quality, ceramic water filtration, household water treatment, recontamination, hygiene

## Abstract

The study assessed changes in water quality between the water source and the tap of locally produced low cost ceramic water filters used by a community living in hygienically critical conditions in a remote mountainous area in Western Nepal. Data was collected from 42 rural households during two visits. The effectiveness of filter handling on its performance was assessed through microbiological analysis, structured household interviews and structured observations. Water quality decreased significantly when source water was filled into transport containers, while the use of the filters improved drinking water quality for about 40% of the households. Highly inadequate filter cleaning practices involving the use of contaminated raw water, hands (geo mean = 110 *E. coli* CFU/100 mL) and cleaning tools (geo mean = 80 *E. coli* CFU/100 mL) stained hygienic parts of the filter. The use of boiling water to disinfect the filters was significantly correlated with improved filter performance and should be further promoted. However, even disinfected filters achieved a very low average LRV for *E. coli* of 0.4 in the field and performed worse than during laboratory tests (LRV for *E. coli* of 1.5–2). Comprehensive training on adequate filter handling, as well as better filter products, are required to improve the impact of filter use.

## 1. Introduction

More than 700 million people worldwide do not have access to an improved source for drinking water and an estimated 1.8 billion people do not consume safe drinking water [1]. In 2010 nearly 1.7 billion cases of diarrhea were caused by the lack of access to safe water, inadequate sanitation and hygiene [2] and 502,000 diarrheal deaths were estimated to be caused by inadequate drinking water [3].

Diarrhea is one of the most common illnesses among children in Nepal and continues to be a major cause of childhood morbidity and mortality. In 2016, diarrhea prevalence among children under the age of 5 was 8% [4]. Precise data on childhood mortality associated with diarrheal diseases in Nepal is not available; however, it has been estimated that approximately 25% of all child deaths are associated with acute diarrhea [5]. Unsafe water and lack of adequate sanitation facilities are important contributing factors to diarrheal diseases in Nepal [6].

The joint monitoring program (JMP) of the World Health Organization (WHO) and United Nations Children’s Fund (UNICEF) reported in 2015 that 92% of the Nepalese population had access to improved water, and hence, met the specific MDG target [7]. However, water has been found to be fecally contaminated in 82% of the households with access to an improved source. Only 14% of the households without improved drinking water use appropriate treatment methods [8].

Household water treatment, if applied correctly and consistently, is a strategy to reduce the health risks related to the consumption of unsafe drinking water [9,10,11,12]. Among different methods for household water treatment, such as solar water disinfection, chlorination and boiling, household water filtration is particularly promising due to its ease of application and high acceptance rate among users [13]. Also, in Nepal, households preferred the application of ceramic water filters when given a choice between different methods [14]. 

Several studies have documented the successful impact of ceramic water filters on improving drinking water quality at the point of consumption in the field context [15], including a reduction of diarrhea in communities using ceramic water filters for drinking water treatment [16,17,18,19]. A study conducted in Kenya found that during six months of use, 71% of filtrate samples collected from locally produced ceramic pot filters contained less than 1 *E. coli* per 100 mL, and although intervention households reported less diarrhea, the difference was not significant [20]. Also, a recently conducted meta-analysis on the impact of drinking water, sanitation and handwashing with soap on childhood diarrhea found that interventions with filters to treat water at the point of consumption together with safe storage reduced diarrhea risk by 61% [12].

Other studies assessed the performance of ceramic water filters on the treatment of water at the point of use in low-income areas and revealed that their use in these settings might not always sufficiently improve drinking water quality. In their household evaluation of ceramic pot filters in Tanzania, Lemons at al. found a moderate filter effectiveness with 42% of the filters reducing *E. coli* to less than 10 CFU/100 mL. The authors related this moderate filter performance to poor manufacturing quality [21]. Lange at al. evaluated the long-term performance of filters with silver-coated ceramic candles that demonstrated more than 4 log removal of coliform bacteria in the lab. After 8 months of use by 51 households in a rural area in South Africa, they found that none of the filtrate samples was free of coliform bacteria and that 35% had higher contamination levels in the filtrate than in the top bucket. Water temperature in the filter above 21 °C negatively influenced the filter’s efficiency, as did the number of children in the household and the frequency that the filter was cleaned [22]. Also, Brown et al. and Mellor et al. have pointed out that improper maintenance of ceramic water filters leads to declining effectiveness in microbiological removal [23,24] 

The lack of supply chains for household water treatment products in remote areas of Nepal is one of the challenges hindering people living in these areas from applying water treatment practices. In the context of the total sanitation campaign launched by the government of Nepal, development organizations working in this field have started to combine behavior change interventions, promoting improved practices for drinking water treatment, sanitation and hygiene with activities to trigger the private sector to make ceramic filters available in more remote areas. As a consequence, locally produced low-cost ceramic candle filters, affordable to local communities, are increasingly sold in rural areas of Nepal. This study was conceived after a water quality and hygiene intervention that promoted these filters in remote areas in Western Nepal did not result in significant water quality improvement at the point of consumption. The goal was to gain a better understanding of water quality changes between the water source, the water transport containers and the tap of ceramic water filters and to assess the influence of critical water and filter handling factors on filter performance and water quality

## 2. Materials and Methods 

### 2.1. Context of the Study

Between 2014 and 2016, Helvetas Swiss Intercooperation implemented a water, sanitation and hygiene intervention in remote hilly areas in Acham, Dailekh, Jajarkot and Kalikot Districts in Western Nepal where the organization previously had installed piped water supply schemes. The intervention consisted of trainings on household water treatment, safe storage, sanitation, handwashing and waste management that took place during three household visits and four group sessions. The project triggered the establishment of supply chains for locally produced low cost ceramic candle water filters and buckets with taps for handwashing in the project areas. The ceramic filters available in the local market consist of two steel bucket containers with one or two ceramic candles in the upper bucket. Different brands of ceramic candles are available that were produced either in India or Nepal: Aqua Fill, Milton, Surya Vinayak and Surya Nepal. These filters are available for 10 to 14 USD, while replacement candles cost 1 to 1.50 USD). None of the candles promoted and sold in the field contained colloidal silver coatings.

The project evaluation, involving 311 randomly selected households, revealed that as a result of the intervention, drinking water treatment increased from 18% at baseline to 86% after the intervention. 77% of the households had purchased a ceramic water filter, 8% used chlorine and 6% boiled water. Water quality analysis revealed that 74% of households had less than 10 *E. coli* CFU/100 mL in their source water, while 5.5% had more than 100 CFU/100 mL. However, at the point of consumption, after collection, transport, treatment and storage (the water was mostly stored in the lower bucket of the ceramic water filter), the water contained less than 10 *E. coli* CFU/100 mL in 47% of the households and more than 100 CFU/100 mL in 22%. In 65% of the households using a ceramic water filter, contamination was higher after filtration than at the source. No deterioration of source water was observed in households using chlorine to treat the water. 91% of the households that tested positive for residual chlorine had an improved water quality [25]. This follow-up study was designed and implemented to better understand the low performance rate of ceramic water filters in the project area and to assess the influence of household hygiene and filter handling practices on water quality changes.

In the project area in Dailekh District, the literacy rate among the main caretakers of children is 44%. 36% have completed informal education, 14.5% primary school, 11.7% secondary school and 9.4% have completed higher education. 89.1% of the households generate their income through agriculture and 40% are female headed households. 31% of these have a husband with foreign employment. 22% of the households produce sufficient food for nine months, 31% for six to nine months and 47% for less than six months. The households in the area do not have access to an electrical grid and use wood for cooking [25].

### 2.2. Laboratory Evaluation of the Ceramic Water Filters

Helvetas evaluated the microbiological removal efficiency and effective life span of all four filter brands promoted in the project area over a period of nine months in a laboratory setting. The removal efficiency of all tested brands declined over time, with a significant drop after the first six months of use. The average Log removal value (LRV) for all brands over a period of nine months was 1.5 (SD = 0.47). Detailed results are presented elsewhere [26]. During another laboratory evaluation, ENPHO found the following LRV’s for *E. coli*: Milton 1.9 (SD = 1.2), Surya Vinayak 2.2 (SD = 1.2) and Surya Nepal 1.8 (SD = 1) [27].

### 2.3. Study Design

Data for the field evaluation was collected during March and April 2017 from 42 rural households using ceramic water filters in Dullu Municipality-3 in Dailekh District through structured observation of water transport and of the cleaning of ceramic water filters, quantitative structured household interviews and water quality and bacteriological analysis. Sample size calculations with G-Power revealed a sample size of 42 households to detect a medium effect of 0.15 at a one-tailed alpha of 0.05 and a statistical power of 80% with multiple linear regression and five predictor variables [28]. The study was conducted in accordance with the Declaration of Helsinki, and the Ethical Review Committee of the Swiss Federal Institute of Aquatic Science and Technology approved the study protocol on 31 December 2016. Households were informed about the goal of the study and the procedure of data collection, and only those households that provided informed consent were involved in the study.

A team of a local and an international researcher accompanied the main person responsible for water management in the household to the main drinking water source (public tap stand for 66.7%, protected source for 21.5% and piped access in the house for 11.8% of households) and conducted a structured observation on how the water users handled the transport container before it was filled at the water source. Water sample WQT1 was taken from the source by letting the water run for some seconds from the tap stand at the water source and then filling 100 mL of water into a sterile Nasco Whirl-Pak. Back at the household, water sample WQT2 was taken from the transport container by pouring 100 mL of water from the container into a sterile Whirl-Pak. Both buckets of the ceramic water filter were emptied, and the upper bucket was filled with water from the transport container. Water sample WQT4 was taken from the tap of the ceramic water filter after filtration. Afterwards, the filter was emptied again and several bacteriological samples were taken from the empty filter. Swab test 1a was taken from the outflow of the ceramic candle by making five circles with the swab from the top to the bottom of the outflow and Swab test 2a was taken from the tap of the ceramic water filter by making five circles with the Nissui Compact Dry cotton swabs from the top to the bottom of the tap of the filter. The swabs were inserted into the test flask containing 1 mL of sterile water solution with 9.0 mg sodium chloride, 12.7 µg potassium dihydrogen phosphate and 78.5 µg disodium phosphate. The flask was shaken for 20 s and the 1 mL solution was poured on Nissui Compact Dry Coli-scan plates and incubated for 24 h at 35 ± 2 °C.

For the bacteriological test BQT1, the filter was taken apart. The lower, now empty bucket of the filter, was directly filled with 500 mL of sterile water. From this volume, 100 mL of water was extracted with a sterile syringe and filled into a sterile Whirl-Pak.

The interviewee was then requested to clean the filter as she normally would do it, and the interviewers followed this process with a structured observation. After cleaning the ceramic filter, the process of taking bacteriological samples from the filter described above was repeated by taking Swab test 1b from the outflow of the ceramic candle, Swab test 2b from the tap of the ceramic water filter and BQT2 from the reservoir of the treated water. After this, the interviewees filled the upper bucket of the filter with water they had previously collected (WQT1). Water sample WQT5 was taken from the tap of the ceramic water filter after filtration.

Next, a bacteriological test (BQT3) was done on the interviewees’ hands. They were requested to first put the right hand into a 2 L Whirl-Pak which was filled with 1 L of sterile water, swirl it for 10 s, and then put the left hand into the same Whirl-Pak and also swirl it for 10 s. For analysis, a 100 mL water sample was extracted from the 2 L Whirl-Pak. If the interviewee used a tool to clean the ceramic water filter, she was requested to put this cloth or sponge into another 2 L Whirl-Pak, which was also filled with 1 L of sterile water. The bag was shaken for 10 s and a 100 mL water sample was extracted to conduct water quality analysis (BQT4).

To assess the contamination of clean water through the filtration process, the interviewers emptied the filter again and filled sterile water into the upper bucket of the ceramic water filter. Water sample WQT6 was taken from the tap of the ceramic water filter after filtration. To assess the performance of clean, not contaminated filters, the filter was emptied and disinfected by filling the upper and lower receptacles with boiling water. The use of chlorinated water was considered as an alternative disinfection option to using boiling water. However, chlorine is not available in the project area and therefore could not be used. Old candles were removed and replaced by new candles. After this, interviewees again filled the upper bucket of the filter with water they had previously collected (WQT1). Water sample WQT7 was collected from the tap of the filter after filtration.

All water samples were kept in Whirl-Paks inside cooler bags and were analyzed at the field site, using membrane filtration techniques. 100 mL water samples were passed through 0.45 µm Millipore cellulose membrane filters, using sterilized filtration equipment. Filter pads were plated on Nissui Compact Dry Coli-scan plates and incubated for 24 h at 35 ± 2 °C. Colonies of total coliforms and *E. coli* were counted.

Quantitative, structured household interviews were conducted after the observed filter cleaning process. The questionnaire contained closed ended, multiple choice questions mostly in categorical variables, but also Likert-scale answer categories and some scale variables. The interviews were complemented by structured observations. The questionnaire was coded in ODK on tablets and contained questions on demographics, the handling of containers used for the transport and storage of water, the operation and maintenance practices of ceramic water filters. Also questions and observations on sanitation and hygiene infrastructure and practices were included as proxy indicators for the hygienic conditions and possible bacterial loads in the household environment which can have impact on water quality during filter handling.

At the end of the household visit, households were trained on adequate operation and maintenance procedures for a ceramic water filter and were given a flyer with instructions. In particular, they were informed that the following parts of the filter should not get into contact with contaminated materials or surfaces: the bottom of the upper bucket, the inside of the lower bucket, the outflow of the candle and the tap of the filter. They were instructed that the candle screws should always be tight. In the case of filter clogging, the candles should be cleaned with a soft brush. Once a week, boiling water should be poured into the upper and the lower buckets of the filter and left for at least 10 min. 

About one week after the first visit, the interviewers visited the households again and requested that the buckets of the filter be emptied and that the upper buckets be refilled with fresh water. The interviewers took the first water sample before filtration and the second sample from the tap of the filter after filtration. How people in the households handled the filter during emptying and refilling was observed and a short quantitative structured interview was conducted on filter operation and maintenance. Households were also asked an open question on how they had cleaned their filters since the first visit. The question was transcribed and coded, and household were grouped into three categories. The category of best practice was attributed if people said that they only used boiled water to clean the inside of the bucket for filtered water, and if they poured boiling water into both buckets and let them stand for at least 10 min. The category of good practice was attributed if people said that they used boiled water to clean the bucket for filtered water. The category of bad practice was attributed if people said that they used untreated water to clean both filter buckets. Five households could not be categorized because the information they provided on their filter cleaning practices was insufficient.

### 2.4. Data Analysis

Data was imported into IBM SPSS Statistics 24 (IBM Corp., Armonk, NY, USA) for statistical analysis. General demographics, container and filter handling factors, as well as sanitation and hygiene infrastructure and practices, were analyzed using descriptive statistics. A two-tailed t-test was used to assess the significance of the differences between Log10 transformed water quality variables. Counts of zero *E. coli* or zero total coliforms were replaced by 0.5 to be able to do logarithmic transformations and calculate geometric means. 

The influence of filter handling on its performance was assessed by correlating filter handling practices and hygiene conditions as stated in the interviews and data on the filter’s contamination levels before the observed cleaning process with the filter’s LRV for *E. coli*. Variables that were significantly correlated with the outcome variable during bivariate analysis were further analyzed by multivariate linear regression. Factors with the lowest significance levels were excluded from the presented model.

## 3. Results

### 3.1. From Source to Transport Container, First Visit

In the study area, the households contain an average of 6.4 (SD = 1.9) people, including 0.8 (SD = 0.9) children that are going to school. A household spends an average of 3 min (SD = 3.2) to collect water from a piped source in the community (67%) or from a protected source (17%). 12% of the households have a piped water connection in their compound. The buckets used to collect water at the source and to transport it home were: narrow necked aluminum (21%), copper (14%) or plastic containers (26%), plastic buckets (31%) and aluminum buckets (5%). 41% of the households covered the water containers with a lid during transport. 

**Figure 1:**
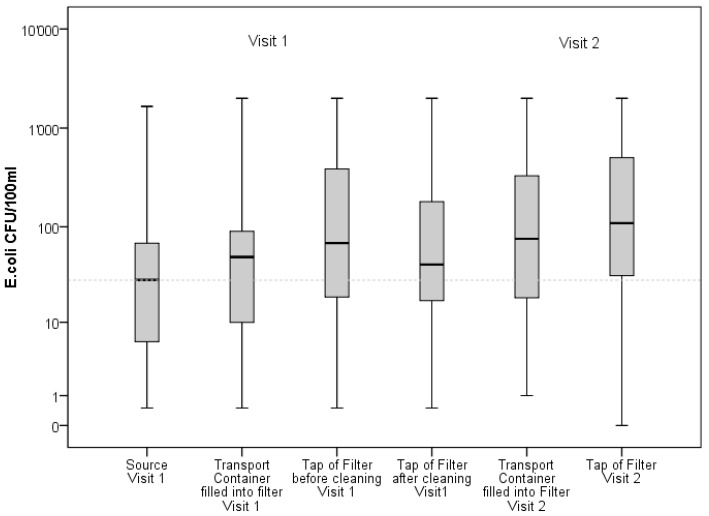
Counts of *E. coli* at the source, in the transport container and at the tap of the ceramic water filter during the first and second household visits.

The filling of water from the source into containers and transporting them led to a statistically significant deterioration of water quality (*t* = −3.09, *p* = 0.004, *r* = 0.43 for Log10 transformed counts of *E. coli*). Counts of *E. coli* increased from a geometric mean of 22 CFU/100 mL (10–90 Percentile: 1–282 CFU/100 mL) at the source to a geometric mean of 41 CFU/100 mL (10–90 Percentile: 3–456 CFU/100 mL) in the transport container (see Figure 1). 

98% of the interviewees said that they clean the container used to transport water every day. Observations revealed that people in all households used raw water to clean the inner side of the transport containers, mainly using their hands (95.2%). Other materials used were soap (16.7%), a soft cloth (16.7%), ash (5%) and a rough cloth (5%). The mean time required to clean the containers was 1.8 min (SD = 1.7). Neither the type of container used, nor any of the observed transport container cleaning factors, was significantly correlated with changes in water quality between water source and the transport container. The counts of *E. coli* after transportation were significantly correlated with the water quality at the source (Pearson’s *r* = 0.7, *p* < 0.001), indicating that higher contamination levels at the source led to higher contamination levels in the transport container.

### 3.2. From Transport Container to Tap of Ceramic Water Filter, First Visit

#### 3.2.1. Water Quality

Water quality measured at the tap of the ceramic filters varied highly among different households. The geometric mean of *E. coli* was 64 CFU/100 mL (10–90 Percentile: 8–954 CFU/100 mL) before the observed cleaning process and 44 CFU/100 mL (10–90 Percentile: 1–945 CFU/100 mL) after it. This difference was not significant (*t* = 1.3, *p* = 0.2).

The quality of water taken from the tap of the ceramic water filter was statistically not different from the water poured into the filter, neither before (*t* = −1.1, *p* = 0.3), nor after the observed filter cleaning process (*t* = −0.2, *p* = 0.8), with mean LRV’s for *E. coli* of −0.2 CFU/100 mL respectively −0.04 CFU/100 mL. The use of the filter before the observed cleaning process improved water quality for 17 households (40%), while the use of the filter after the observed cleaning process improved water quality for 21 households (50%). Figure 1 presents counts of *E. coli* at the source, in the transport container, at the tap of the ceramic water filter before and after the observed cleaning process, and at the tap of the ceramic water filter during the second household visit.

**Figure 2:**
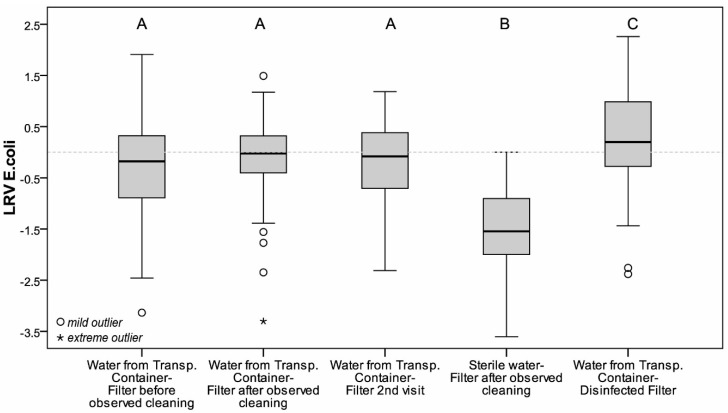
Log removal values of contaminated and disinfected household filters: (**A**) water from the transport container passed through a contaminated filter; (**B**) sterile water passed through a contaminated filter and (**C**) water from the transport container passed through a disinfected filter.

Sterile water passed through the filter after the observed cleaning process contained a geometric mean of *E. coli* of 18 CFU/100 mL (10–90 Percentile: 2–187 CFU/100 mL) after filtration. Figure 2 shows that the filters contaminated the sterile water with a mean LRV for *E. coli* of −1.6 (SD = 0.8). When water from the transport container was filled into the filters that had been disinfected by the researchers and equipped with new candles, the household filters achieved a mean LRV for *E. coli* of 0.42 (SD = 1.2). Water passed through such filters contained a geometric mean of *E. coli* of 8 CFU/100 mL.

Figure 2 shows that, although the use of disinfected filters slightly improved water quality, the use of non-disinfected filters did not improve the quality of water for more than half of the households. In all households, sterile water was contaminated by passing it through the filter.

#### 3.2.2. Filter Handling 

During the interview, 64% of the households stated that they had not received any instructions on filter operation and maintenance from the seller of the filter and the filter cleaning practices of the households were observed to be highly inadequate. People in all households used raw water and their hands to clean the filters, while a majority used a cloth to wash the hygienic parts of the filter. 5% of the households said that they use boiled water to wash the lower bucket of the filter. Peoples’ hands and the cloths used for cleaning were both highly contaminated, with a geometric mean of *E. coli* of 110 CFU/100 mL (10–90 Percentile: 6–2000 CFU/100 mL) on hands and 80 CFU/100 mL (10–90 Percentile: <1–2000 CFU/100 mL) on the cloths. Details on filter handling and hygiene practices are presented in Table S1 in the supporting materials. 

Bacteriological tests on sterile water poured into the lower bucket of the filters found a geometric mean of 30 *E. coli* CFU/100 mL (10–90 Percentile: 1–1940 CFU/100 mL) before the observed cleaning process. The geometric mean was 70 *E. coli* CFU/100 mL 10–90 Percentile: 2–2000 CFU/100 mL) after the observed cleaning process. Swab tests on the outflow of the ceramic candle and the tap of the filter did not detect any *E. coli*.

Observation of the filter cleaning process during the first visit revealed that all households took the two buckets of the filter apart for cleaning. During the cleaning process, 83% of the households placed critical parts of the filter, such as the outflow of the ceramic candle (65%), the bottom of the raw water reservoir (48%), the inner side of the clean water reservoir (35%) and the tap of the clean water reservoir (45%), on contaminated surfaces or touched it with contaminated materials. 80% of the households used soap to clean the outside of the filters, while 31% used soap to clean the inner side of the lower filter buckets. 5% of the households used boiled water to clean the lower filter buckets. The actual process of filter cleaning was predicted well during the interview, but we assume that a slightly more rigorous approach was applied under observation such as the use of more water for cleaning and longer as well as more thorough rubbing of the different parts.

The following factors were significantly correlated with the filter’s LRV before the observed cleaning process in bivariate analysis:

- Candles had been replaced (Pearson’s *r* = 0.73, *p* = 0.021)
- Slippers (shoes) are available in the toilet (Pearson’s *r* = 0.4, *p* = 0.011)
- Boiled water is used to clean the filter (Pearson’s *r* = 0.396, *p* = 0.009)
- Amount of water collected daily by the household (Pearson’s *r* = 0.347, *p* = 0.025
- The frequency of filter cleaning (Pearson’s *r* = 0.312, *p* = 0.044)
- Contamination level in the lower bucket of the filter (Pearson’s *r* = −0.564, *p* < 0.001)
- A soft cloth is used for the daily cleaning of the filter (Pearson’s *r* = −0.457, *p* = 0.002)
- A soft cloth is used for the daily cleaning of the transport container (Pearson’s *r* = −0.436, *p* = 0.004)

Except for the availability of slippers (shoes) in the toilet, none of the factors used as proxy indicators for household cleanliness was significantly correlated with the filters’s LRV (type of toilet in the household, cleanliness of the toilet, availability of a brush in the toilet, availability of water to flush the toilet, availability and type of handwashing facilities in the household, condition of the handwashing facilities, availability of soap and water at the handwashing facilities, frequency of hand washing per day). The availability of slippers (shoes) in the toilet indicates that the household generally had a higher awareness on the importance of hygienic practices. Also, the variable was positively correlated with the availability of piped water in the household (χ^2^(1) = 5.09, *p* = 0.024), a factor which again supports hygiene. Households with slippers (shoes) in the toilet were more likely to report that they had not received instructions on filter handling (χ^2^(1) = 8.5, *p* = 0.004) and they were less likely to use soap for the cleaning of filters (χ^2^(1) = 5.5, *p* = 0.019).

As presented in Table 1, a multivariate linear regression model with the filter’s LRV before the observed cleaning process as outcome variable revealed that the performance of the filter could be significantly improved by the use of boiled water during filter cleaning. Households with slippers (shoes) in the toilet had higher LRV’s, indicating that general sanitary hygiene conditions influence the filter’s effectiveness. The filter’s performance was negatively affected if the filters had higher contamination levels of *E. coli* in the lower bucket (the reservoir for the filtered water) and if the households said that they used a soft cloth for the daily cleaning of the transport container.

#### 3.2.3. From Transport Container to Tap of Ceramic Water Filter, Second Visit

The observation of filter emptying and refilling during the second household visit revealed that when emptying the filter, 62% of the households touched the inside of the lower filter bucket (the reservoir for the filtered water), 21% touched the bottom of the upper bucket and 7% touched the outflow of the candles. During the interviews that took place at this visit, people said that they had cleaned the filter an average of 2.3 days ago (SD = 1.8). Materials used to clean the filter were said to be: a brush (74%), raw water (69%), boiled water (43%), soap (55%), a soft cloth (16.7%) or a rough cloth (5%). 76.2% of the households were able to show the instruction flyer for filter maintenance that they had received during the first project visit.

When asked about important elements of filter operation and maintenance, people in the households highlighted the following points: use boiled water to wash the filter (78.6%), clean candle with a soft brush (71.4%), do not touch the bottom of the raw water bucket, do not touch the inside of the filtered water bucket (50%), pour boiling water into the filter and let it stand for 10 min (50%), assure that the screw of the candle is tight (42.9%), do not touch the outflow (28.6%), and do not use soap or ash to clean the filter (28.6%).

40.5% of the households received a very good categorization on the basis of their account on how they clean the filters and 5% a good practice categorization. The practices of 43% of the households were rated as bad. 12% of the households could not be categorized.

The above shows that the short training session achieved a slight, but still insufficient change in awareness on best practices in filter operation and maintenance. Only half of the households were aware that they should not touch the inside of the filtered water bucket and should use boiling water to disinfect it. Also, the change in awareness achieved by the training did not sufficiently result in adequate change in their practices.

Water samples taken from the transport containers, as well as from the tap of the ceramic water filters during the second household visit, had a higher geometric means of *E. coli* than during the first household visit, with 74 CFU/100 mL (10–90 Percentile: 7–1560 CFU/100 mL) in the transport container and 97 CFU/100 mL (0–90 Percentile: 10–1207 CFU/100 mL) at the tap of the filter. However, the difference of log-transformed counts of these samples were statistically not significant from the sample taken from the tap before the observed cleaning process during the first visit (*t* = −1.57, *p* = 0.12 (see Figure 1). Log-transformed counts of *E. coli* in water filled into the filter were not significantly different from the log-transformed counts of *E. coli* in water collected at the tap of the filter (*t* = −0.87, *p* = 0.4). The filter’s LRV measured during the second visit did not differ from the LRV measured during the first visit (see Figure 2).

**Figure 3:**
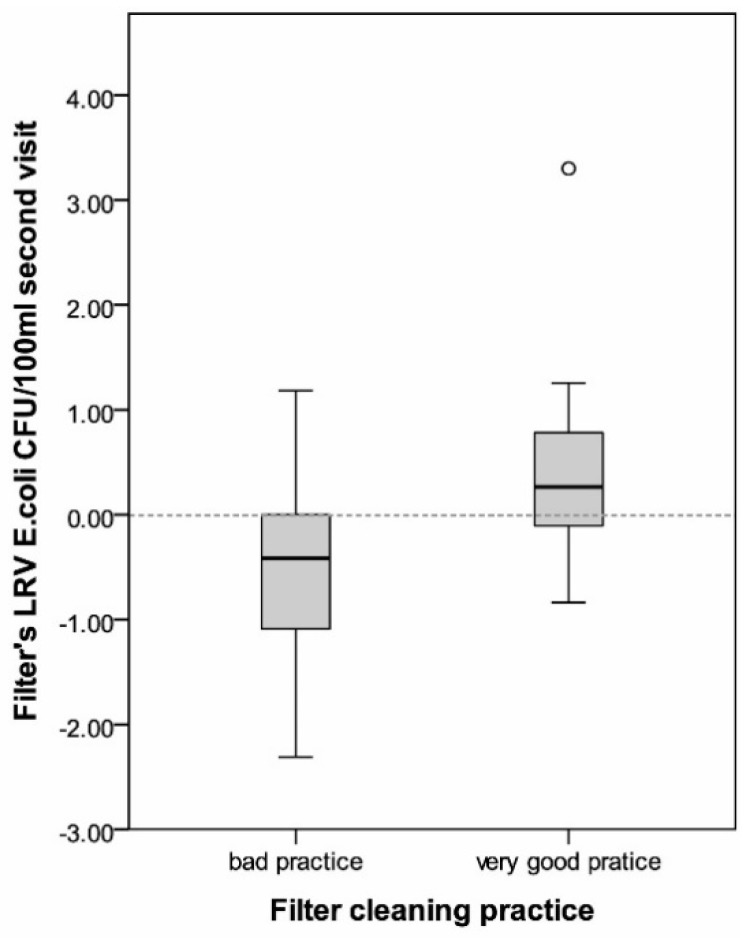
Filter performance in households with very good and bad cleaning practices.

However, variability within different households was high and the household’s filter cleaning practices significantly correlated with the filter’s LRV of *E. coli* (Pearson’s *r* = 0.32, *p* = 0.04). Households that were attributed a very good cleaning categorization achieved a slight improvement in drinking water quality with a mean LRV = 0.4 CFU/100 mL (SD = 0.9). Water in households with a bad practice attribution was further contaminated by the filtration process with LRV= −0.5 CFU/100 mL (SD = 0.8) (see Figure 3).

The use of a ceramic water filter improved the quality of water collected at the source for 16 households (40%), while it further contaminated water for 24 households (60%). LRV’s higher than 1 were only achieved by three households. The percentage of households with deteriorated water quality (41%) and with improved water quality (17%) was identical during the first and second household visits. 

The findings on the general performance of well disinfected filters during the second household (LRV = 0.4) visit are supported by the findings from the first household visit. The researchers found that the ceramic filters had a very low performance even after disinfection, with a mean LRV for *E. coli* of 0.42 CFU/100 mL. A similar rate of disinfection was achieved by households that were attributed a very good cleaning categorization that involved disinfection with boiling water.

## 4. Discussion

Our study was conducted in a very remote mountainous area in Nepal with relatively high water quality at the source (geometric mean = 22 *E. coli* CFU/100 mL) in local communities with high poverty levels and critical hygiene conditions. After water was filled from the source into transport containers, there was a significant deterioration in drinking water quality. Since neither the type of transport containers used, nor any of the practices applied for cleaning transport containers before filling them with water, were associated with the change in water quality, it is probable that the handling of the container in the household would account for the contamination.

The use of the locally produced ceramic water filters improved drinking water quality for 40% of the households during both visits, but led to a decrease in water quality for the other households. Sterile water passed through the filter was contaminated with a mean LRV for *E. coli* of −1.6. This indicates that the ceramic filters used by the local communities were highly contaminated with fecal coliforms, leading to deterioration of water quality during filter use for a majority of users. This is not surprising as we observed highly inadequate filter handling practices, such as the use of raw water, hands that were highly contaminated (geometric mean = 110 *E. coli* CFU/100 mL) and cloths that were also highly contaminated (80 *E. coli* CFU/100 mL), to clean hygienic parts of the filter. The regression analysis revealed that regular cleaning of the filter with a soft cloth was significantly associated with water contamination during filter use.

Previous studies have documented the significant impact ceramic water filter interventions had on improving drinking water quality and, subsequently, reducing health risks [15,16,17,19,20,29]. However, contrary to our expectations, this intervention did not achieve a satisfactory improvement in drinking water quality at the point of consumption. This is partly due to poor filter handling practices and use in an environment with low hygiene and high loads of fecal bacteria in the households. Yet, households had access to source water of quite high quality and, therefore, maximally obtainable LRV’s were low. 40.5% of the households were attributed a very good cleaning categorization which included the use of boiling water to regularly disinfect the filter. These households achieved a LRV for *E. coli* of 0.4.

The use of filters of low quality contributed to their low performance in the field. Filters previously used by households that were disinfected by researchers before collecting the water sample at the filter’s tap achieved a LRV of only 0.42–identical to households that were attributed a good cleaning categorization. This is significantly lower than the disinfection efficiency of the same filter brands obtained during laboratory analysis, which was LRV of 1.5–2 [26,27], and indicates that the extended use of these filters by local households in the field reduced the quality of the filter candles. It was also observed that users regularly brushed candles, sometimes extensively, to obtain higher flow rates, which can lead to the removal of ceramic layers from the candle to an extent that even a few holes were found in some of the candles. 

To achieve a stronger impact and to better improve drinking water quality at the point of consumption, future interventions should place more emphasis on adequate handling of ceramic water filters. Improved practice should consist of the regular use of boiling water to disinfect the filters. Raw water, and hands or cloths should not be used to clean hygienic parts of the filter. Using a brush to clean the filter candle should only happen when the candle is blocked. Another finding was that doing a short training event was not sufficient to achieve significant changes in awareness and better practices in filter operation and maintenance. Comprehensive behavior change strategies should be implemented to properly establish adequate practices [30].

In addition to training proper filter handling, providing users access to filters of higher quality can significantly improve the impact of water quality interventions. This has been documented in several studies that evaluated the effectiveness of household membrane filters in field settings [31,32,33]. The introduction of higher priced ceramic water filters into the markets of poor and remote mountainous areas, such as the region where our intervention took place, however, faces challenges. People in the local communities mostly lack the resources to pay for higher priced filters. Filters adequate for use in such settings should fulfill quality as well as pricing criteria [34].

### Limitations

The swab tests used to identify bacterial contamination on the outflow of the ceramic candle and the tap of the filter did not detect coliforms. However, it could be that our methodological approach of making five circles with the cottons swabs was not sufficient to detect sufficient coliforms attached to the inner surface of the filter’s outflow and the filter’s tap.

## 5. Conclusions

Water quality significantly deteriorated after the source water was filled into transport containers. This indicates that the first steps to reduce the contamination of drinking water should target the containers used for drinking water transport to assure that they are hygienic. The use of ceramic water filters improved drinking water quality in 40% of the households. Yet, due to contamination issues their use lead to further deterioration of water quality in the other households.

A short training led to a slight, yet insufficient, increase in awareness on improved filter handling, but was not sufficiently translated into practice. More than half of the households still practiced inadequate filter cleaning practices after the training, i.e., the use of peoples’ hands and untreated water to clean the lower bucket of the filter where the filtered water is stored. The use of boiling water to regularly clean and disinfect the filters was identified as an important factor that enhanced filter performance. The practice of regularly using boiling water for filter disinfection should, therefore, be established in communities using ceramic water filters for water disinfection. Comprehensive training and education would be necessary to strengthen the required behavior change. 

The locally produced ceramic water filters used in the project area achieved a very low performance in the field. Even when boiling water was used for disinfection, the average LRV for *E. coli* was 0.4. Filters performed worse in the field than during laboratory tests (LRV for *E. coli* of 1.5–2). It is probable that the practice of extensively brushing filter candles to achieve a higher flow rate could have damaged filter candles to such an extent that some had holes in them. Households, therefore, should be trained to not regularly use soft brushes to clean filter candles. These should be used only when the candles are getting clogged. In addition, better performing, but still low-cost, household filters are needed in the field to achieve higher disinfection rates in a scenario where households have good filter operation and maintenance practices. Our study indicates that promotion campaigns for ceramic water filters in remote areas with critical hygienic conditions require filter products of sufficient quality as well as adequate training on operation and maintenance to assure that water quality at the tap of the filters is safe for consumption.

## Figures and Tables

**Table 1:** Linear regression model: Factors influencing the filter’s LRV.

Model: Dependent Variable: LRV *E. coli* (*E. coli* in the Transport Container— *E. coli* at the Tap of the Ceramic Water Filter)				
	B	SE (B)	Beta	*p*
Use of boiled water to clean the filter	1.19	0.43	0.300 **	0.009
Availability of slippers (shoes) in the toilet	0.70	0.29	0.280 *	0.021
Daily cleaning of the transport container with a soft cloth	−0.66	0.30	−0.270 *	0.037
Contamination level of *E. coli* in lower bucket (reservoir for filtered water)	0.00	0.00	−0.300 *	0.016
Cleaning of the filter with a soft cloth	−0.58	0.30	−0.24	0.062
(Constant)	0.08	0.22		0.718

*R*^2^ = 0.63, * *p* < 0.05, ** *p* < 0.01.

## Supplementary Materials

The following are available online at http://www.mdpi.com/1660-4601/15/11/2439/s1, Table S1: Frequencies-Interview & Observation: Water use, handling transport container, handling ceramic water filter, Use and condition of hygiene infrastructure.
