# Supplementary material for: From Water Source to Tap of Ceramic Filters—Factors That Influence Water Quality Between Collection and Consumption in Rural Households in Nepal
Source: Int J Environ Res Public Health. 2018 Nov 1;15(11):2439. doi: 10.3390/ijerph15112439 (PMC6266981; doi:10.3390/ijerph15112439)
Supplement: Supplementary file 1 [file ijerph-15-02439-s001.pdf]

Table S1

Frequencies: Interview & Observation:

Water use, handling transport container, handling ceramic water filter, Use and condition of hygiene infrastructure

	N		Percentag	Median	Mean	Std. Deviation
	Valid	Missing				
People in the household	42	0		6.000	5.881	1.8637
Nr. of school going children in household	42	0		1.000	.762	.8782
Time required to collect water	42	0		2.000	3.000	3.1623
Amount of water collected per day in L	42	0		56.000	61.143	22.6173
Main water source used to collect drinking water						
piped source at household level	42		11.9			
piped source in the village	42		66.7			
protected source (well, spring)	42		16.7			
dangling pipe	42		4.80			
**Observation: use and cleaning of transport container**						
Container used to collect drinking water today						
Gagri aluminium	42	0	21.40			
Plastic bucket	42	0	21.40			
Gagri copper	42	0	16.70			
Jerrycan plastic	42	0	28.60			
Other container	42	0	11.90			
Containers with a lid	42	0	40.5			
Materials used to clean the transport container						
soft cloth	42	0	16.7			
hands	42	0	95.2			
soap	42	0	16.7			
raw water	42	0	100			
ash	42	0	4.8			
rough cloth	42	0	4.8			
Mean time required to clean the transport container (Min.)	42	0			1.83	1.7
Clean transport container everyday	42	0	97.60			
Do not have extra storage container available	40	2	95.00			
Use a filter since Nr. of months	42	0		14.000	19.190	10.0468
Have replaced candle	40	2	27.50			
Candlereplacement Nr of months ago	11	31		6.00	5.09	3.477
Candle_age (months)	42	0		12.0000	15.2381	10.59178
Have received an instruction on filter O&M (yes)	42	0	35.70			
Frequency of filling the filter per day	42	0		2.000	2.095	.5763
Candles are well screwed into the filter	42	0	70			
Frequency of cleaning the filter						
clean filter_once per week	42	0	50.00			
clean filter_every 3rd day	42	0	28.60			
clean filter_every 2nd day	42	0	7.10			
clean filter_every day	42	0	14.30			
Frequency of washing hands with soap per day	42	0		4.000	4.214	2.2145
**Observation: Cleaning of ceramic water filters**						
Unscrew candle for cleaning	42	0	4.8			
Pour water out from clean water reservoir	42	0	58.5			
Clean parts touch dirty surface	42	0	**82.9**			
Bottom of raw water reservoir touches dirty surface	42	0	47.5			
Outflow of ceramic water candle touches dirty surface	42	0	65			
Inside of clean water reservoir touches dirty tool	42	0	35			
Tap touches dirty surface or dirty tool	42	0	45			
Materials used to clean the filter outside						
soft cloth	42	0	41			
rough cloth	42	0	12.8			
brush	42	0	2.6			
hands	42	0	**97.4**			
soap	42	0	79.5			
raw water	42	0	**94.9**			
boiled water	42	0	5.1			
chlorine	42	0	0			
ash	42	0	5.1			
earth	42	0	0			
Materials used to clean the raw water reservoir inside						
soft cloth	42	0	22			
rough cloth	42	0	0			
brush	42	0	22			
hands	42	0	**95.1**			
soap	42	0	31.7			

raw water	42	0	**95.1**			
boiled water	42	0	4.9			
chlorine	42	0	0			
ash	42	0	4.9			
earth	42	0	0			
Materials used to clean the candle						
soft cloth	42	0	7.3			
rough cloth	42	0	0			
brush	42	0	48.8			
hands	42	0	**78**			
soap	42	0	19.5			
raw water	42	0	**90.2**			
boiled water	42	0	12.2			
chlorine	42	0	0			
ash	42	0	0			
earth	42	0	0			
Materials used to clean the clean water reservoir inside						
soft cloth	42	0	23			
rough cloth	42	0	2.6			
brush	42	0	12.8			
**hands**	42	0	**94.9**			
soap	42	0	30.8			
**raw water**	42	0	**89.7**			
**boiled water**	42	0	5.1			
chlorine	42	0	0			
ash	42	0	2.6			
earth	42	0	0			
Materials used to clean the tap of the filter						
soft cloth	42	0	31.4			
rough cloth	42	0	2.8			
brush	42	0	13.9			
hands	42	0	75			
soap	42	0	**41.7**			
raw water	42	0	**86.1**			
boiled water	42	0	5.6			
chlorine	42	0	0			
ash	42	0	5.6			
earth	42	0	0			
Materials used to clean the lid of the filter						
soft cloth	42	0	44.4			
rough cloth	42	0	11.1			
brush	42	0	8.3			
hands	42	0	**100**			
soap	42	0	**72.2**			
raw water	42	0	**94.4**			
boiled water	42	0	5.6			
chlorine	42	0	0			
ash	42	0	5.6			
earth	42	0	0			
Hands touch clean parts of the filter during the cleaning process			100			
**Observation: Hygiene conditions**						
Household saying that they are boiling drinking water	42	0	4.80			
Chlorine for drinking water treatment available	42	0	0.00			
Filter for drinking water treatment available	42	0	100.00			
Filter candle has cracks	42	0	4.80			
Water sealed toilet in household	42	0	97.60			
Pitlatrine in household	42	0	2.40			
Condition of toilet						
Toilet is clean (no visible traces of feaces)	42	0	100.00			
Cleaning brush is available in toilet	40	2	90.00			
Water to flush available in toilet	40	2	97.50			
Cleaning soap available in toilet	40	2	65.00			
Slippers available in toilet	40	2	35.00			
Type of handwashing facilities in household						
bucket with a tap	42	0	90.50			
piped handwashing station	42	0	4.80			
no handwashing station	42	0	4.80			
Condition of handwashing facilities						
handwashing facilities in good condition (no damages)	40	2	95.00			
soap available next to handwashing facilities	40	2	87.50			
handwashing facilities clean	40	2	90.00			
water available next to handwashing facilities	40	2	97.50			
